# 1q21.1 distal copy number variants are associated with cerebral and cognitive alterations in humans

**DOI:** 10.1038/s41398-021-01213-0

**Published:** 2021-03-22

**Authors:** Ida E. Sønderby, Dennis van der Meer, Clara Moreau, Tobias Kaufmann, G. Bragi Walters, Maria Ellegaard, Abdel Abdellaoui, David Ames, Katrin Amunts, Micael Andersson, Nicola J. Armstrong, Manon Bernard, Nicholas B. Blackburn, John Blangero, Dorret I. Boomsma, Henry Brodaty, Rachel M. Brouwer, Robin Bülow, Rune Bøen, Wiepke Cahn, Vince D. Calhoun, Svenja Caspers, Christopher R. K. Ching, Sven Cichon, Simone Ciufolini, Benedicto Crespo-Facorro, Joanne E. Curran, Anders M. Dale, Shareefa Dalvie, Paola Dazzan, Eco J. C. de Geus, Greig I. de Zubicaray, Sonja M. C. de Zwarte, Sylvane Desrivieres, Joanne L. Doherty, Gary Donohoe, Bogdan Draganski, Stefan Ehrlich, Else Eising, Thomas Espeseth, Kim Fejgin, Simon E. Fisher, Tormod Fladby, Oleksandr Frei, Vincent Frouin, Masaki Fukunaga, Thomas Gareau, Tian Ge, David C. Glahn, Hans J. Grabe, Nynke A. Groenewold, Ómar Gústafsson, Jan Haavik, Asta K. Haberg, Jeremy Hall, Ryota Hashimoto, Jayne Y. Hehir-Kwa, Derrek P. Hibar, Manon H. J. Hillegers, Per Hoffmann, Laurena Holleran, Avram J. Holmes, Georg Homuth, Jouke-Jan Hottenga, Hilleke E. Hulshoff Pol, Masashi Ikeda, Neda Jahanshad, Christiane Jockwitz, Stefan Johansson, Erik G. Jönsson, Niklas R. Jørgensen, Masataka Kikuchi, Emma E. M. Knowles, Kuldeep Kumar, Stephanie Le Hellard, Costin Leu, David E. J. Linden, Jingyu Liu, Arvid Lundervold, Astri Johansen Lundervold, Anne M. Maillard, Nicholas G. Martin, Sandra Martin-Brevet, Karen A. Mather, Samuel R. Mathias, Katie L. McMahon, Allan F. McRae, Sarah E. Medland, Andreas Meyer-Lindenberg, Torgeir Moberget, Claudia Modenato, Jennifer Monereo Sánchez, Derek W. Morris, Thomas W. Mühleisen, Robin M. Murray, Jacob Nielsen, Jan E. Nordvik, Lars Nyberg, Loes M. Olde Loohuis, Roel A. Ophoff, Michael J. Owen, Tomas Paus, Zdenka Pausova, Juan M. Peralta, G. Bruce Pike, Carlos Prieto, Erin B. Quinlan, Céline S. Reinbold, Tiago Reis Marques, James J. H. Rucker, Perminder S. Sachdev, Sigrid B. Sando, Peter R. Schofield, Andrew J. Schork, Gunter Schumann, Jean Shin, Elena Shumskaya, Ana I. Silva, Sanjay M. Sisodiya, Vidar M. Steen, Dan J. Stein, Lachlan T. Strike, Ikuo K. Suzuki, Christian K. Tamnes, Alexander Teumer, Anbupalam Thalamuthu, Diana Tordesillas-Gutiérrez, Anne Uhlmann, Magnus O. Ulfarsson, Dennis van ‘t Ent, Marianne B. M. van den Bree, Pierre Vanderhaeghen, Evangelos Vassos, Wei Wen, Katharina Wittfeld, Margaret J. Wright, Ingrid Agartz, Srdjan Djurovic, Lars T. Westlye, Hreinn Stefansson, Kari Stefansson, Sébastien Jacquemont, Paul M. Thompson, Ole A. Andreassen, for the ENIGMA-CNV working group, Dennis van der Meer, Eco J. C. de Geus, Greig I. de Zubicaray, Sonja M. C. de Zwarte, Stephanie Le Hellard, Dennis van ‘t Ent, Marianne B. M. van den Bree

**Affiliations:** 1 NORMENT, Division of Mental Health and Addiction, Oslo University Hospital and Institute of Clinical Medicine, University of Oslo, Oslo, Norway; 2 Department of Medical Genetics, Oslo University Hospital, Oslo, Norway; 3 KG Jebsen Centre for Neurodevelopmental Disorders, University of Oslo, Oslo, Norway; 4 School of Mental Health and Neuroscience, Faculty of Health, Medicine and Life Sciences, Maastricht University, Maastricht, the Netherlands; 5 Sainte Justine Hospital Research Center, Montreal, Quebec, Canada; 6 Centre de recherche de l’Institut universitaire de gériatrie de Montréal, Montreal, Quebec, Canada; 7 Department of Psychiatry and Psychotherapy, University of Tübingen, Tübingen, Germany; 8 deCODE Genetics (Amgen), Reykjavík, Iceland; 9 Faculty of Medicine, University of Iceland, Reykjavík, Iceland; 10 Department of Clinical Biochemistry, Copenhagen University Hospital, Rigshospitalet, Glostrup Denmark; 11 Department of Psychiatry, Amsterdam UMC, University of Amsterdam, Amsterdam, the Netherlands; 12 Department of Biological Psychology and Netherlands Twin Register, VU University Amsterdam, Amsterdam, the Netherlands; 13 University of Melbourne Academic Unit for Psychiatry of Old Age, Kew, Australia; 14 National Ageing Research Institute, Parkville, Australia; 15 Institute of Neuroscience and Medicine, INM-1, Research Centre Jülich, Jülich, Germany; 16 C. and O. Vogt Institute for Brain Research, Medical Faculty, University Hospital Düsseldorf, Heinrich Heine University Duesseldorf, Düsseldorf, Germany; 17 Umeå Centre for Functional Brain Imaging, Umeå University, Umeå, Sweden; 18 Department of Integrative Medical Biology, Umeå University, Umeå, Sweden; 19 Mathematics and Statistics, Murdoch University, Perth, Australia; 20 Research Institute, Hospital for Sick Children, Toronto, Ontario Canada; 21 South Texas Diabetes and Obesity Institute, Department of Human Genetics, School of Medicine, University of Texas Rio Grande Valley, Brownsville, USA; 22 Amsterdam Neuroscience, Amsterdam, the Netherlands; 23 Amsterdam Public Health Research Institute, VU Medical Center, Amsterdam, the Netherlands; 24 Centre for Healthy Brain Ageing, School of Psychiatry, University of New South Wales, Sydney, Australia; 25 Dementia Centre for Research Collaboration, School of Psychiatry, University of New South Wales, Sydney, Australia; 26 Department of Psychiatry, University Medical Center Brain Center, Utrecht University, Utrecht, the Netherlands; 27 Institute of Diagnostic Radiology and Neuroradiology, University Medicine Greifswald, Greifswald, Germany; 28 Altrecht Science, Utrecht, the Netherlands; 29 Tri-institutional Center for Translational Research in Neuroimaging and Data Science (TReNDS), Georgia State University, Georgia Institute of Technology, Emory University, Atlanta, USA; 30 The Department of Electrical and Computer Engineering, University of New Mexico, Albuquerque, USA; 31 Institute for Anatomy I, Medical Faculty, Heinrich Heine University Düsseldorf, Düsseldorf, Germany; 32 Imaging Genetics Center, Mark and Mary Stevens Institute for Neuroimaging and Informatics, University of Southern California, Los Angeles, USA; 33 Department of Biomedicine, University of Basel, Basel, Switzerland; 34 Institute of Medical Genetics and Pathology, University Hospital Basel, Basel, Switzerland; 35 Department of Psychosis Studies, Institute of Psychiatry, Psychology and Neuroscience, King’s College London, London, United Kingdom; 36 University Hospital Marqués de Valdecilla, IDIVAL, Centro de Investigación Biomédica en Red Salud Mental (CIBERSAM), Santander, Spain; 37 University Hospital Virgen del Rocío, IBiS, Centre de Investigació Biomédica en Red Salud Mental (CIBERSAM), Sevilla, Spain; 38 Center for Multimodal Imaging and Genetics, University of California, San Diego, USA; 39 Department of Psychiatry and Neuroscience Institute, University of Cape Town, Cape Town, Western Cape South Africa; 40 Department of Psychological Medicine, Institute of Psychiatry, Psychology and Neuroscience, King’s College London, London, United Kingdom; 41 Faculty of Health, Queensland University of Technology, Brisbane, Australia; 42 Social, Genetic & Developmental Psychiatry Centre, Institute of Psychiatry, Psychology and Neuroscience, King’s College London, London, United Kingdom; 43 MRC Centre for Neuropsychiatric Genetics and Genomics, Cardiff University, Cardiff, United Kingdom; 44 Cardiff University Brain Research Imaging Centre School of Psychology, Cardiff University, Cardiff, United Kingdom; 45 Centre for Neuroimaging and Cognitive Genomics, School of Psychology and Discipline of Biochemistry, National University of Ireland Galway, Galway, Ireland; 46 Laboratory for Research in Neuroimaging LREN, Centre for Research in Neurosciences, Department of Clinical Neurosciences, Lausanne University Hospital and University of Lausanne, Lausanne, Switzerland; 47 Neurology Department, Max-Planck-Institute for Human Cognitive and Brain Sciences, Leipzig, Germany; 48 Division of Psychological and Social Medicine, Faculty of Medicine, TU Dresden, Dresden, Germany; 49 Language and Genetics Department, Max Planck Institute for Psycholinguistics, Nijmegen, the Netherlands; 50 Department of Psychology, University of Oslo, Oslo, Norway; 51 Bjørknes College, Oslo, Norway; 52 Signal Transduction, H. Lundbeck A/S, Ottiliavej 9, DK-2500 Valby, Denmark; 53 Donders Institute for Brain, Cognition and Behaviour, Radboud University, Nijmegen, the Netherlands; 54 Department of Neurology, Akershus University Hospital, 1474 Nordbyhagen, Norway; 55 Institute of Clinical Medicine, Campus Ahus, University of Oslo, Oslo, Norway; 56 Université Paris-Saclay, CEA, Neurospin, 91191 Gif-sur-Yvette, France; 57 Division of Cerebral Integration, National Institute for Physiological Sciences, Okazaki, Japan; 58 Department of Life Science, Sokendai, Hayama, Japan; 59 Psychiatric and Neurodevelopmental Genetics Unit, Center for Genomic Medicine, Massachusetts General Hospital, Boston, MA USA; 60 Department of Psychiatry, Massachusetts General Hospital, Harvard Medical School, Boston, MA USA; 61 Stanley Center for Psychiatric Research, Broad Institute of MIT and Harvard, Cambridge, MA USA; 62 Boston Children’s Hospital, Boston, Massachusetts USA; 63 Institute of Living, Hartford, Connecticut USA; 64 Harvard Medical School, Boston, Massachusetts USA; 65 Department of Psychiatry and Psychotherapy, University Medicine Greifswald, Greifswald, Germany; 66 German Center of Neurodegenerative Diseases (DZNE), Rostock/Greifswald, Greifswald, Germany; 67 Department of Biomedicine, University of Bergen, Bergen, Norway; 68 Division of Psychiatry, Haukeland University Hospital, Bergen, Norway; 69 Department of Neuromedicine and Movement Science, Norwegian University of Science and Technology, Trondheim, Norway; 70 St Olav’s Hospital, Department of Radiology and Nuclear Medicine, Trondheim, Norway; 71 School of Medicine, Cardiff University, Cardiff, United Kingdom; 72 Department of Pathology of Mental Diseases, National Institute of Mental Health, National Center of Neurology and Psychiatry, Kodaira, Japan; 73 Osaka University, Osaka, Japan; 74 Princess Màxima Center for Pediatric Oncology, Utrecht, the Netherlands; 75 Genentech, Inc., South San Francisco, 94080 CA USA; 76 Department of Child and Adolescent Psychiatry/Psychology, Erasmus MC-Sophia, Rotterdam, the Netherlands; 77 Institute of Human Genetics, University of Bonn Medical School, Bonn, Germany; 78 Psychology Department, Yale University, New Haven, CT USA; 79 Department of Psychiatry, Yale University, New Haven, CT USA; 80 Department of Psychiatry, Massachusetts General Hospital, Boston, MA USA; 81 Interfaculty Institute for Genetics and Functional Genomics, University Medicine Greifswald, Greifswald, Germany; 82 Department of Psychiatry, Fujita Health University School of Medicine, Toyoake, Japan; 83 Department of Clinical Science, University of Bergen, Bergen, Norway; 84 Department of Medical Genetics, Haukeland University Hospital, Bergen, Norway; 85 Centre for Psychiatry Research, Department of Clinical Neuroscience, Karolinska Institutet, & Stockholm Health Care Services, Stockholm Region, Stockholm, Sweden; 86 Norwegian Centre for Mental Disorders Research (NORMENT), Institute of Clinical Medicine, University of Oslo, Oslo, Norway; 87 Department of Clinical Biochemistry, Copenhagen University Hospital Rigshospitalet, Glostrup, Denmark; 88 Department of Clinical Medicine, Faculty of Health and Medical Sciences, University of Copenhagen, Copenhagen, Denmark; 89 Department of Genome Informatics, Graduate School of Medicine, Osaka University, Osaka, Japan; 90 Norwegian Centre for Mental Disorders Research, Department of Clinical Science, University of Bergen, Bergen, Norway; 91 Dr Einar Martens Research Group for Biological Psychiatry, Department of Medical Genetics, Haukeland University Hospital, Bergen, Norway; 92 Department of Clinical and Experimental Epilepsy, UCL Queen Square Institute of Neurology, London, WC1N 3BG UK; 93 Genomic Medicine Institute, Lerner Research Institute, Cleveland Clinic, Cleveland, Ohio, United States; 94 Chalfont Centre for Epilepsy, Chalfont-St-Peter, United Kingdom; 95 Mohn Medical Imaging and Visualization Centre, Department of Radiology, Haukeland University Hospital, Bergen, Norway; 96 Department of Biological and Medical Psychology, University of Bergen, Bergen, Norway; 97 Service des Troubles du Spectre de l’Autisme et apparentés, Lausanne University Hospital, Lausanne, Switzerland; 98 Genetic Epidemiology, QIMR Berghofer Medical Research Institute, Brisbane, Australia; 99 Neuroscience Research Australia, Randwick, Australia; 100 Herston Imaging Research Facility and School of Clinical Sciences, Queensland University of Technology, Brisbane, Australia; 101 Institute for Molecular Bioscience, University of Queensland, Brisbane, Australia; 102 Queensland Brain Institute, University of Queensland, Brisbane, Australia; 103 Psychiatric Genetics, QIMR Berghofer Medical Research Institute, Brisbane, Australia; 104 Department of Psychiatry and Psychotherapy, Central Institute of Mental Health, Medical Faculty Mannheim, University of Heidelberg, Mannheim, Germany; 105 University of Lausanne, Lausanne, Switzerland; 106 Department of Radiology and Nuclear Medicine, Maastricht University Medical Center, Maastricht, the Netherlands; 107 School for Mental Health and Neuroscience, Maastricht University, Maastricht, the Netherlands; 108 Institute of Psychiatry, Psychology and Neuroscience, King’s College London, London, United Kingdom; 109 The CatoSenteret Rehabilitation Center, Son, Norway; 110 Department of Radiation Sciences, Umeå University, Umeå, Sweden; 111 Center for Neurobehavioral Genetics, University of California, Los Angeles, USA; 112 Department of Psychiatry, Erasmus University Medical Center, Rotterdam, The Netherlands; 113 Bloorview Research Institute, Holland Bloorview Kids Rehabilitation Hospital, Toronto, Ontario, Canada; 114 Physiology and Nutritional Sciences, University of Toronto, Toronto, Ontario, Canada; 115 Departments of Radiology and Clinical Neurosciences, University of Calgary, Calgary, Alberta Canada; 116 Bioinformatics Service, Nucleus, University of Salamanca, Salamanca, Spain; 117 Centre for Population Neuroscience and Precision Medicine, Institute of Psychiatry, Psychology and Neuroscience, King’s College London, London, United Kingdom; 118 Department of Psychosis, Institute of Psychiatry, Psychology & Neuroscience, Kings College, London, United Kingdom; 119 Psychiatric Imaging Group, MRC London Institute of Medical Sciences (LMS), Hammersmith Hospital, Imperial College, London, United Kingdom; 120 Institute of Psychiatry, Psychology and Neuroscience, London, London, United Kingdom; 121 Neuropsychiatric Institute, The Prince of Wales Hospital, Sydney, Australia; 122 University Hospital of Trondheim,Department of Neurology and Clinical Neurophysiology, Trondheim, Norway; 123 Neuroscience Research Australia, Sydney, Australia; 124 School of Medical Sciences, University of New South Wales, Sydney, Australia; 125 Institute of Biological Psychiatry, Roskilde, Denmark; 126 The Translational Genetics Institute (TGEN), Phoenix, AZ United States; 127 Department of Human Genetics, Radboud University Medical Center, Nijmegen, the Netherlands; 128 South African Medical Research Council Unit on Risk and Resilience in Mental Disorders, Department of Psychiatry and Neuroscience Institute, University of Cape Town, Cape Town, South Africa; 129 VIB Center for Brain & Disease Research, Stem Cell and Developmental Neurobiology Lab, Leuven, Belgium; 130 University of Brussels (ULB), Institute of Interdisciplinary Research (IRIBHM) ULB Neuroscience Institute, Brussels, Belgium; 131 The University of Tokyo, Department of Biological Sciences, Graduate School of Science, Tokyo, Japan; 132 PROMENTA Research Center, Department of Psychology, University of Oslo, Oslo, Norway; 133 Department of Psychiatry, Diakonhjemmet Hospital, Oslo, Norway; 134 Institute for Community Medicine, University Medicine Greifswald, Greifswald, Germany; 135 Department of Radiology, Marqués de Valdecilla University Hospital, Valdecilla Biomedical Research Institute IDIVAL, Santander, Spain; 136 Faculty of Electrical and Computer Engineering, University of Iceland, Reykjavík, Iceland; 137 VIB-KU Leuven Center for Brain & Disease Research, 3000 Leuven, Belgium; 138 KU Leuven, Department of Neurosciences & Leuven Brain Institute, 3000 Leuven, Belgium; 139 Université Libre de Bruxelles (U.L.B.), Institut de Recherches en Biologie Humaine et Moléculaire (IRIBHM), and ULB Neuroscience Institute (UNI), 1070 Brussels, Belgium; 140 National Institute for Health Research, Mental Health Biomedical Research Centre, South London and Maudsley National Health Service Foundation Trust and King’s College London, London, United Kingdom; 141 Centre for Advanced Imaging, University of Queensland, Brisbane, Australia; 142 Department of Pediatrics, University of Montreal, Montreal, Quebec, Canada

**Keywords:** Psychiatric disorders, Clinical genetics, Molecular neuroscience

## Abstract

Low-frequency 1q21.1 distal deletion and duplication copy number variant (CNV) carriers are predisposed to multiple neurodevelopmental disorders, including schizophrenia, autism and intellectual disability. Human carriers display a high prevalence of micro- and macrocephaly in deletion and duplication carriers, respectively. The underlying brain structural diversity remains largely unknown. We systematically called CNVs in 38 cohorts from the large-scale ENIGMA-CNV collaboration and the UK Biobank and identified 28 1q21.1 distal deletion and 22 duplication carriers and 37,088 non-carriers (48% male) derived from 15 distinct magnetic resonance imaging scanner sites. With standardized methods, we compared subcortical and cortical brain measures (all) and cognitive performance (UK Biobank only) between carrier groups also testing for mediation of brain structure on cognition. We identified positive dosage effects of copy number on intracranial volume (ICV) and total cortical surface area, with the largest effects in frontal and cingulate cortices, and negative dosage effects on caudate and hippocampal volumes. The carriers displayed distinct cognitive deficit profiles in cognitive tasks from the UK Biobank with intermediate decreases in duplication carriers and somewhat larger in deletion carriers—the latter potentially mediated by ICV or cortical surface area. These results shed light on pathobiological mechanisms of neurodevelopmental disorders, by demonstrating gene dose effect on specific brain structures and effect on cognitive function.

## Introduction

Inter-individual differences in brain structure are highly heritable^1^, but identifying the genes that contribute to brain development is challenging. Genome-wide association studies (GWAS) of brain anatomical structures indicate the influence of many single-nucleotide polymorphisms (SNPs) with small effect sizes^2,3^, but the links to brain function remain weak. Evidence is emerging that some rare copy number variants (CNVs)—that is, regions of the genome that are either deleted or duplicated—are associated with both substantial brain size and shape differences; for example, the 7q11.23^4,5^, 22q11.2^6,7^, 15q11.2^8–11^ and 16p11.2 proximal^12–14^ and distal CNVs^15^. Many of these CNVs also have a wide-ranging phenotypic impact, including poorer cognitive abilities^8,16–18^ and increased risk of neurological or neurodevelopmental disorders. The strong impact of these CNVs on brain structure and behaviour make them valuable for studies of the molecular mechanisms contributing to aberrant human neurodevelopment.

The 1q21.1 distal CNV has a known large effect on head circumference, as evident from a high prevalence of micro- and macrocephaly in deletion and duplication carriers, respectively^19–21^. This, along with its position in a region that is rich in genes unique to the human lineage (i.e. absent in primates)^22,23^, makes the 1q21.1 distal CNV particularly interesting for the study of aberrations in human brain structure. However, its relatively low frequency, 1 in ~3400, (deletions) and 1 in 2100 (duplications)^8,16^, has hampered the study of its effects on brain structure.

1q21.1 distal deletion and duplication carriers are both at higher risk for several neurodevelopmental disorders including schizophrenia^24,25^, intellectual disability (ID), developmental delay, speech problems, autism spectrum disorders, motor impairment^19,26–28^ and epilepsy^26,29^, in addition to the separate risk for the duplication carriers for ADHD^30^, bipolar disorder and major depression^31,32^. Further, general cognitive ability (IQ) was lower in carriers in a small clinical study^19^ and in the UK Biobank^33^. In addition, 1q21.1 distal CNVs display a positive dose response on head circumference^19–21^, height and weight^34,35^ and are associated with various somatic diseases and traits, including bone and muscle deviations^34^ and cataract^36^ (deletion only), diabetes^36^ (duplication only) and heart disease^36–39^ (both). Conversely, several studies report carriers without any clinically evident phenotypes^19,38^ and considerable heterogeneity^40,41^, suggesting incomplete penetrance and variable expressivity. The Df(h1q21)+/− mouse, deleted in the syntenic 1q21.1 distal region, displays some phenotypes similar to human CNV carriers, including reduced head-to-tail length and altered dopamine transmission in response to psychostimulants, as seen in people with schizophrenia^42^.

The 1q21 region in humans is rich in low copy number repeats^20,43^ and contains several recurrent CNVs with differing breakpoints^21,37^. Thus, gene estimates vary, but the core interval encompasses at least 12 protein-coding genes including several human-specific genes such as *HYDIN2*^21,37^, *NOTCH2NL*s^22,23^ and the DUF1220/Olduvain domain-containing *NBPF*-encoding genes^44–46^—the two latter were recently shown to have evolved as a two-gene unit^47^. Particularly interesting in the context of brain development are the recently characterized *NOTCH2NL* genes, absent in human’s closest living relatives and shown to prolong cortical neurogenesis^22,23^.

Despite the strong effects on neurodevelopmental traits and disorders, the impact of the 1q21.1 CNVs on human brain structure is largely unknown. Here, we present the first large-scale systematic neuroimaging study of 1q21.1 distal CNV carriers, investigating brain structure in >37,000 individuals including 28 deletion and 22 duplication carriers. We mapped the effect of the 1q21.1 distal CNV on subcortical volumes, intracranial volume (ICV) and global and regional measures of mean cortical thickness and surface area. We investigated variation in cognitive task performance and supplemented with exploratory mediation analysis of the brain on cognition in the UK Biobank. Given prior findings^19–21,48^, we explored a dose-dependent effect of copy number on brain structures and decreased cognitive performance for both 1q21.1 distal deletion and duplication carriers in comparison to non-carriers.

## Materials and methods

### Sample description

The brain structural sample comprises a total of 39 cohorts with genotyping and magnetic resonance imaging (MRI) data—38 from the ENIGMA-CNV consortium in addition to a subsample of the UK Biobank^49^ (project ID #27412). Demographic characteristics for each cohort are described in Supplementary Table 1 with a reference to participants’ collection and datasets including individual inclusion and exclusion parameters. Extended information on diagnosis and family information can be found in Supplementary Note 1 and age distribution of the cohorts in Supplementary Fig. 1. All participants gave written informed consent and sites involved obtained ethical approvals. The main 1q21.1 distal sample consisted of 28 deletion carriers, 22 duplication carriers and 37,088 non-carriers (Table 1) from 13 different datasets and 15 scanner sites with various ascertainments (family, clinical and population studies, case–control study for psychiatric disease) collected up until 30 September 2019. Non-carriers were defined as having no CNVs known to cause neurodevelopmental diseases (as defined in Supplementary Table 2). In the meta-analysis, an independent Icelandic sample from deCODE Genetics consisting of two deletion carriers and five duplication carriers in addition to 1150 non-carriers was added.

**Table 1:** Demographic data.

	ENIGMA-CNV				deCODE			
	del	nc	dup	*P*	del	nc	dup	*P*
*n*	28	37,088	22		2	1150	5	
Sex, male (%)	15 (54%)	17,912 (48%)	9 (41%)		1 (50%)	511 (44%)	2 (33%)	
Age (mean (SD))	41.7 (19.0)	61.1 (12.8)	55.4 (12.7)	<0.001	53.5 (2.1)	44.8 (12.4)	46.4 (16.5)	
Children (age <18 years)	4 (14%)	665 (1.8%)		<0.001	0	0	0	
Known diagnosis (%)	11 (39.3%)	2424 (6.5%)	7 (32%)	<0.001		238 (21%)	2 (40%)	
Disease type (%)								
ADHD		1 (~0%)				181 (16%)	2 (40%)	
Autism						2 (0.2%)		
Bipolar disorder						7 (0.6%)		
Clinically recruited (no diagnosis)	6 (21.4%)		4 (18%)					
Dyslexia	1 (3.6 %)							
F-ICD-10 diagnosis (UK Biobank)		858 (2.3%)	1 (4%)					
G-ICD10 diagnosis (UK Biobank)	1 (3.0%)	1439 (3.8%)	1 (4%)					
MDD		1 (~0%)						
Multiple diagnoses^a^	2 (7.2%)		1 (4.5%)					
Persistent depressive disorder		1 (~0%)						
SCZ	1 (3.6)	124 (0.3)				48 (4.2%)		
Scanner sites	11	15	8		2	2	1	
Datasets	9	13	7		1	1	1	

*ADHD* attention deficit disorder, clinically recruited in clinical NDD study but without a diagnosis, *MDD* major depressive disorder, *SCZ* schizophrenia, *del* deletion carrier, nc non-carriers, *dup* duplication carrier, *P* *P* value, *AvPD* avoidant personality disorder, *OCD* obsessive-compulsive disorder, DPD dependent personality disorder, *STPD* schizotypal personality disorder, *NS* non-significant.

*P* value is based on a *χ*^2^ test for categorical values and ANOVA for continuous values.

^a^First deletion carrier: agoraphobia, AvPD, OCD, DPD, other substance-related disorder, conduct disorder. Second deletion carrier: specific phobia, social phobia, MDD, AvPD, STPD. Duplication carrier: social phobia, OCD, MDD, AvPD.

### Genotyping and QC

The genotypes were obtained by genotyping with commercially available platforms, performed at participating sites for each cohort (Supplementary Table 1). Individuals were excluded exclusively based on quality control (QC) parameters from the CNV calling. No exclusion was done due to ancestry in the primary analysis, but the effect of ancestry was evaluated in a separate analysis (see below).

### CNV calls and validation in the core ENIGMA-CNV sample

Almost all cohorts had CNVs called and identified in a unified manner as described previously^15^. In brief, CNVs were called using PennCNV^50^ and appropriate population frequency (PFB) files and GC (content) model files (Supplementary Table 3 and Supplementary Notes2 and 3). Samples were filtered and CNVs identified based on standardized QC metrics^15^ (Supplementary Notes 2 and 3). The 1q21.1 distal region was well covered by all arrays (Supplementary Fig. 2). CNVs overlapping the region of interest (1q21.1 distal and 1q21.1 distal and proximal) were identified with the R package iPsychCNV, visualized and manually inspected.

### Image acquisition and processing

All brain measures were obtained from structural T1-weighted MRI data collected at participating sites around the world and analysed with the standardized image analysis, FreeSurfer, quality assurance and statistical methods as per the harmonized neuroimaging protocols developed within ENIGMA2^3^ and ENIGMA3 (http://enigma.ini.usc.edu/protocols/imaging-protocols/). Further detail on data processing is provided in Supplementary Note 4. Details on study, scanner, vendor, field strength, sequence, acquisition parameters and FreeSurfer versions used are outlined in Supplementary Table 4.

### Statistical analysis

Imaging data processing and CNV calling were performed locally and de-identified CNV and imaging data were provided for a central mega-analysis. One of a pair of duplicates was kept. Relatives were removed from the sample used for the main analysis. In addition, we conducted a number of sensitivity analyses to test the robustness of the results (Supplementary Note 5 and Supplementary Tables 5–8). Individuals with a minimum overlap of 0.4 to regions with known pathogenic CNVs (Supplementary Table 2) were excluded from the analysis regardless of copy number status as were individuals from scanner sites without 1q21.1 distal CNV carriers.

Brain measures were normalized in R v3.3.2 by an inverse normal transformation of the residual of a linear regression on the phenotype correcting for covariates as done previously^15^. For the primary analysis, covariates were age, age^2^, sex, scanner site and ICV. In the analysis of ICV, ICV was not included as a covariate. These final covariance-corrected values were used in downstream analysis and are reported for each measure. For comparison between groups, normalization was carried out including only the groups addressed (deletion and non-carriers, duplications and non-carriers) except for the deletion versus duplication comparison, where values from normalization of the entire dataset were used due to the low numbers.

For the copy number dosage effect analysis (i.e. the effect on brain structure of 1q21.1 distal copy number variation), a linear regression on the copy number status of the individuals (deletion = 1, normal = 2, duplication = 3) was performed using the following model: covariance-corrected, normalized brain measure ~ copy number (deletion = 1, non-carrier = 2, duplication = 3). For comparison between groups, a two-sample, two-sided *t* test assuming equal variance in all carrier/non-carrier groups was employed (R v3.3.2) where deletion or duplication carriers were compared either to each other or to non-carriers. To correct for the multiple comparisons, we calculated the number of independent outcome measures through the spectral decomposition of a correlation matrix using MatSpDlite (https://neurogenetics.qimrberghofer.edu.au/matSpDlite/) of the three global, seven subcortical and 68 regional cortical measures. Based on the ratio of observed eigenvalue variance to its theoretical maximum, the estimated equivalent of independent measures was 36. Thus, we set the significance threshold at *α* = 0.05/36 = 0.0014. We report the uncorrected *P* values throughout the manuscript.

Effect size is calculated as the absolute effect size (the difference in mean between the two copy number groups in the *t* test—which, in this case, equals Cohen’s *D* as the standard deviation of the normalized brain measures is one) and the estimate of beta in the linear regression. Plots were generated using R library ggplot2 v2.2.1^51^. Regional cortical visualization was done with the R package ggseg v1.5.1.

In a novel analysis, the independent Icelandic data were processed and analysed as the main dataset. We meta-analysed the results using the R package *metafor* v2.0.0, as previously^15^.

#### Cognitive task performance data

We downloaded behavioural performance measures on seven cognitive tests (the pairs matching task, the reaction time task, reasoning and problem-solving tests, the digit span test, the symbol digit substitution test and the trail making A and B tests) from the UK Biobank repository, performed by at least 10% of the participants. The results were processed following the general approach by Kendall et al.^16^. For more details, see Supplementary Note 6. For the analysis of the seven cognitive measures, we set the significance threshold to *α* = 0.05/7 = 0.007.

### Mediation analysis

Mediation analyses were done with the R package *mediation* v4.4.7. Brain measures were normalized as described above and cognitive tasks were corrected for age, age^2^ and sex prior to input into the analysis. We report the proportion of the total effect of the CNV on cognitive task performance mediated by the brain measures (‘path ab’/‘path c’), with *P* values calculated through quasi-Bayesian approximation using 5000 simulations. We set the significance threshold at *α* = 0.05/((2 + 4) × 6) = 1.4 × 10^−3^ given the test of two structures for deletion and four for duplication carriers on six cognitive tests. The digit span test was excluded since no 1q21.1 CNV carriers had results from both this cognitive test and brain structural data.

## Results

### Sample characteristics

The main 1q21.1 distal (146.5–147.4 Mb, hg19) brain structural dataset consisted of 28 deletion and 22 duplication carriers and 37,088 non-carriers (derived from the same scanner sites as the CNV carriers) from ENIGMA-CNV and UK Biobank (Table 1, separate demographics in Supplementary Table 9). The age of CNV carriers was lower (41.7 ± 19.0 (deletions), 55.4 ± 12.7 (duplications), respectively) than that of non-carriers (61.1 ± 12.1) (Table 1). Eleven deletion carriers and seven duplication carriers had a known neurological, neurodevelopmental or psychiatric diagnosis or had been recruited in a clinical CNV study. The remaining carriers either did not have an established diagnosis or were recruited in studies from which diagnostic information was unavailable (Table 1 and Supplementary Table 10). Of the 37,088 non-carriers, 6.5% (2425) had an established neurological, neurodevelopmental or psychiatric disorder.

### 1q21.1 distal CNV associated with global cortical surface structures

**Fig. 1:**
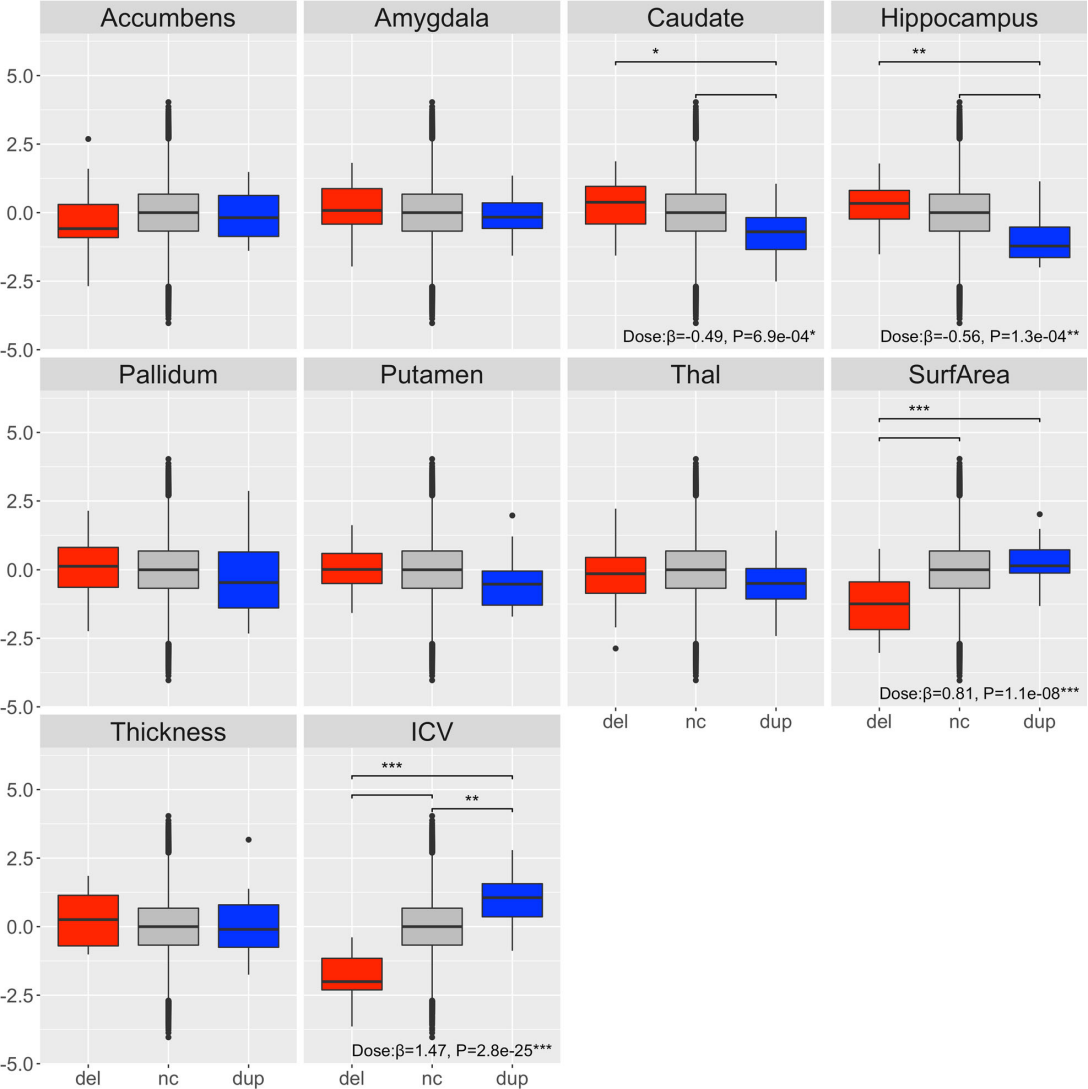
Cortical surface area and ICV show a positive dosage effect and caudate and hippocampus a negative dosage effect to copy number in the 1q21.1 distal region in our main sample (ENIGMA-CNV and UK Biobank). Boxplots of subcortical volumes, cortical surface area and mean cortical thickness and ICV are shown. Deletion carriers (del) in red, non-carriers (nc) in grey and duplication carriers (dup) in blue, respectively. The normalized brain values are presented. Boxplots represent the mean. Copy number dosage effect is noted at the bottom of each panel. Significant differences after correction between groups are noted as *P < 0.0014, **P < 0.00014, ***P = 0.000014. Centre line represents the median, box limits are the upper and lower 25% quartiles, whiskers the 1.5 interquartile range and the points are the outliers. All analyses were corrected for age, age squared, sex, scanner site and ICV (except for ICV).

For our main dataset, there was a significant positive association between the number of 1q21.1 distal copies and ICV (*β* = 1.47, *P* = 2.8 × 10^−25^) as well as cortical surface area (*β* = 0.81, *P* = 1.1 × 10^−8^) (Fig. 1 and Supplementary Table 5) at a significance threshold of *P* < 0.0014 after correction for age, age^2^, sex, scanner site and ICV. In contrast, a significant negative copy number dosage effect was identified for the caudate (*β* = −0.49, *P* = 6.9 × 10^−4^) and hippocampal volumes (*β* = −0.56, *P* = 1.3 × 10^−4^). *T* tests indicated a decrease in ICV (Cohen’s *D* = −1.84 (−17%), *P* = 1.6 × 10^−22^) for deletion carriers and an increase for duplication carriers (Cohen’s *D* = 0.90 (+10%), *P* = 2.3 × 10^−5^), respectively, compared to non-carriers (Supplementary Table 6). For a raw value plot of ICV, see Supplementary Fig. 3. The cortical surface area dosage effect was primarily driven by the deletion carriers with a significantly lower total cortical surface area (Cohen’s *D* = −1.13 (−23%), *P* = 2.1 × 10^−9^) and the dosage effect on caudate and hippocampus was primarily driven by duplication carriers with significantly smaller caudate (Cohen’s *D* = −0.71 (−16%), *P* = 0.0012) and hippocampal (Cohen’s *D* = −0.92 (−15%), *P* = 4.1 × 10^−5^) volumes than non-carriers (Fig. 1 and Supplementary Table 7). Adding an independent Icelandic dataset with two deletions, five duplications and 1150 non-carriers (Table 1) in a meta-analysis strengthened the majority of the dosage results (Supplementary Fig. 4 and Supplementary Tables 11 and 12) and revealed additional significant between-group differences in nucleus accumbens, caudate and putamen (Supplementary Table 12).

A number of sensitivity analyses were run on the main dataset, namely:

- Matching each carrier with one non-carrier for age, sex, scanner site and ICV or age, sex, scanner site;
- including only: (i) non-affected individuals (i.e. excluding individuals with a known neurodevelopmental or neurological disorder diagnosis; (ii) adults (age ≥ 18); (iii) non-affected adults; (iv) children (age < 18); (v) ENIGMA-CNV or (vi) UK Biobank;
- controlling for ancestry;
- excluding ICV as a covariate or;
- including first- and second-degree relatives (see Supplementary Note 5 for methods).

These analyses validated the overall effects (Supplementary Tables 5 and 6).

### The 1q21.1 distal CNV is associated with regional brain structures

**Fig. 2:**
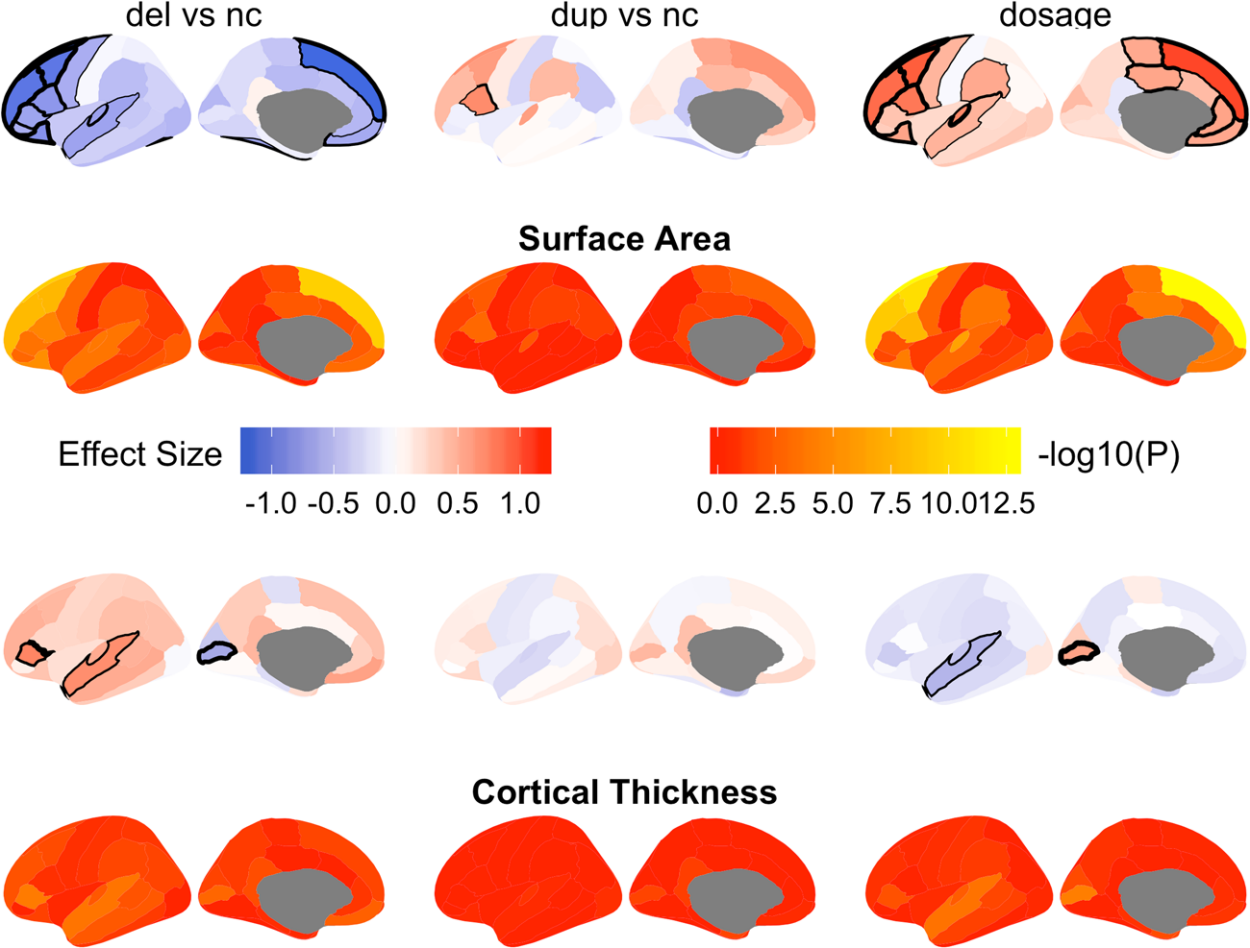
Results from the *t* tests and linear regression of 1q21.1 copy number variation on regional cortical surface area and cortical thickness. First and third rows: Effect sizes (Cohen’s *d* for the *t* tests, beta coefficient for the dosage/linear regression). Second and fourth rows: Statistical significance in –log 10 of the *P* value. Significant areas in rows 1 and 3 are marked with black lines with increasing thickness for increasing significance (*P* < 0.0014). The column names indicate the comparisons with del = deletion carriers, nc = non-carriers, dup = duplication carriers. All measures were corrected for age, age^2^, sex, scanner site and ICV.

The largest dosage effects for the regional cortical surface area were found in the frontal lobes followed by the cingulate cortex—with additional significant effects in three regions of the parietal and temporal lobes (Fig. 2 and Supplementary Table 7). Likewise, through *t* tests, the largest effects in both deletion and duplication carriers in comparison to non-carriers were observed in the frontal and cingulate cortices (Fig. 2 and Supplementary Table 8).

For regional cortical mean thickness, we identified significant negative dosage effects in the superior temporal region and significant positive dosage effects for the pericalcarine region (Fig. 2 and Supplementary Tables 7 and 8). Similarly, significant increases in mean cortical thickness were observed in deletion carriers versus non-carriers in the pars triangularis and superior temporal regions and a significant decrease in the pericalcarine region (Fig. 2 and Supplementary Table 8). All regional results were corrected for age, age^2^, sex, scanner site and ICV. Sensitivity analyses similar to those performed for subcortical regions confirmed the robustness of the results (Supplementary Tables 7 and 8).

### 1q21.1 distal CNV associated with cognitive performance and mediation by brain structures

Deletion and duplication carriers had different cognitive profiles in comparison to non-carriers when testing for association in seven different neuropsychological tests available from the full UK Biobank sample: deletion carriers had significantly poorer performance in three tests: symbol digit substitution, trail making B and pairs matching, while duplication carriers had significantly poorer performance in two tests: reaction time and the reasoning and problem-solving task (Table 2).

**Table 2:** 1q21.1 CNV deletion and duplication carriers show deficits in specific cognitive functions.

Test	Suggested domain	*n*			del vs. nc		dup vs. nc	
		del	nc	dup	Cohen’s *D* (SE)	*P*	Cohen’s *D* (SE)	*P*
Pairs matching	Working memory	119	468,709	186	**−0.36 (0.09)**	**7.3E** **−** 05**	0.03 (0.01)	0.7
Reaction time	Simple processing speed	115	464,648	181	−0.12 (0.06)	0.18	**−0.23 (0.07)**	**2.1E** **−** **03**
Reasoning and problem solving	Fluid intelligence	29	154,490	71	−0.48 (0.19)	9.2E − 03	**−0.33 (0.12)**	**5.3E** **−** **03**
Digit span	Numeric memory	12	47,569	27	−0.27 (0.14)	0.36	0.14 (0.07)	0.47
Symbol digit substitution	Complex processing speed	24	111,900	28	**−0.78 (0.2)**	**1.4E** **−** 04**	0.04 (0.02)	0.83
Trail making A	Visual attention	23	98,495	27	−0.29 (0.15)	0.16	−0.14 (0.07)	0.45
Trail making B	Visual attention	23	98,494	27	**−0.87 (0.21)**	**3.1E** **−** 05***	−0.19 (0.1)	0.33

*n* sample size, *del* deletion carriers, *dup* duplication carriers, *nc* non-carriers, *SE* standard error, *P* *P* value.

Multiple comparison-corrected significant findings (*P* < 0.007) are indicated in bold and with *<0.007, **<0.0007 and ***<0.00007.

Testing the effect of brain structures on cognitive tests in UK Biobank participants, larger ICV and total surface area were associated with better performance on almost all tests (Table 3 and see Supplementary Table 13 for sample size details). A larger hippocampus was associated with better performance for symbol digit substitution, trail making A and B (Table 3) and a larger caudate was associated with higher performance on the trail making A (Table 3).

**Table 3:** Mediation analysis of brain structures over the association between 1q21.1 distal CNV carrier status and performance in the cognitive tasks in the UK Biobank.

	Path B—effect of brain structure on cognition		Deletion		Duplication	
	Estimate (SE)	*P*	Prop. mediated	*P*	Prop. mediated	*P*
Pairs matching						
Caudate	0.0023 (0.0053)	0.66			3.5E − 03	0.85
Hippocampus	0.005 (0.0052)	0.34			9.8E − 03	0.68
SurfArea	**0.031 (0.0055)**	**1.9E** **−** **08**	−0.07	0.65	−4.4E − 03	0.9
ICV	**0.027 (0.0054)**	**4.3E** **−** **07**	−0.12	0.64	−0.07	0.51
Reaction time						
Caudate	−0.0016 (0.0054)	0.77			−2.3E − 03	0.67
Hippocampus	0.01 (0.0053)	0.053			0.01	0.04
SurfArea	−0.0095 (0.0056)	0.091	0.02	0.13	7.3E − 04	0.78
ICV	0.029 (0.0055)	**2.4E** **−** **07**	−0.1	0.07	**−0.03**	**2.4E** **−** **03**
Reasoning and problem solving						
Caudate	−0.0059 (0.0091)	0.51			5.7E − 03	0.55
Hippocampus	0.0031 (0.0089)	0.73			−9.6E − 05	0.95
SurfArea	**0.052 (0.0094)**	**2.6E** **−** **08**	0.06	0.250	−7.4E − 04	0.97
ICV	**0.15 (0.0092)**	**3.7E** **−** **59**	0.25	0.24	0.18	0.04
Symbol digit substitution						
Caudate	0.0011 (0.0077)	0.88			−4.2E − 03	0.83
Hippocampus	**0.04 (0.0075)**	**6.5E** **−** **08**			−0.01	0.82
SurfArea	**0.055 (0.0079)**	**3.8E** **−** **12**	**0.05**	**2.4E** **−** **03**	6.9E − 04	0.99
ICV	**0.066 (0.0079)**	**3.6E** **−** **17**	**0.1**	**4.0E** **−** **04**	0.13	0.68
Trail making A						
Caudate	**0.034 (0.0084)**	**5.7E** **−** **05**			4.4E − 04	1
Hippocampus	**0.04 (0.0081)**	**1.0E** **−** **06**			3.0E − 03	0.97
SurfArea	**0.046 (0.0086)**	**1.1E** **−** **07**	0.09	0.19	1.1E − 03	0.98
ICV	**0.059 (0.0085)**	**6.1E** **−** **12**	0.21	0.20	−0.01	0.99
Trail making B						
Caudate	0.021 (0.0083)	0.012			−0.01	0.79
Hippocampus	**0.04 (0.008)**	**6.9E** **−** **07**			−0.01	0.86
SurfArea	**0.082 (0.0085)**	**6.4E** **−** **22**	**0.07**	**8.0E** **−** **04**	8.9E − 03	0.92
ICV	**0.11 (0.0084)**	**1.2E** **−** **36**	**0.17**	**1.2E** **−** **03**	0.16	0.73

Path B is the effect of the brain structure on cognition overall including all 1q21.1 deletion and duplication carriers (4–13 CNV carriers in each group) and non-carriers (*n* = 10,501–30,924; for exact numbers, see Supplementary Table 13). Each calculation included 5000 simulations.

The significance value for multiple comparisons (1.4 × 10^−3^) are in bold

Next, we tested whether the brain structures significantly associated with 1q21.1 distal CNV carriers might mediate the effect of the CNV on cognition. For two of the three tests associated with deletion carrier status, there were significant mediation effects (significance threshold 1.4 × 10^−3^): cortical surface area and ICV accounted for 5 and 10%, respectively, of the poorer performance of deletion carriers on symbol digit substitution, and 7 and 17%, respectively, of their poorer performance on the trail making B test (Table 3).

## Discussion

Our main finding was a significant positive dosage effect in humans of 1q21.1 distal copy number on ICV and cortical surface area, with the largest differences in frontal and cingulate cortical surface area. We also identified a significant negative dosage effect on caudate and hippocampal volumes. A number of sensitivity analyses confirmed the robustness of the results. Both 1q21.1 distal deletion and duplication carriers showed poorer cognitive performance, although on different tests, with an indication that decreased ICV/cortical surface area might mediate the effect in deletion carriers.

### The 1q21.1 distal CNV causes copy dosage effect on brain structures

We found a strong effect of the 1q21.1 distal CNV on the total cortical surface area, while no overall effect on mean cortical thickness was observed. A specific increase in the size of the cortical surface area with little effect on cortical thickness is observed throughout mammalian evolution including the primate lineage leading to humans^52^. This possibly reflects that cortical thickness and surface area appear to be driven by distinct genetic processes^53^. This pattern may be the result of an increased number of symmetric or self-renewing cell-division cycles, leading to an expansion of the neural progenitor pool and subsequently to an increase in the number of cortical neurons—in line with the radial unit hypothesis^52^. Interestingly, although not significant, mean cortical thickness tended to decrease in deletion carriers in the frontal cortical surface areas with the highest effect sizes, resembling a pattern found in lissencephaly^54^. This could suggest that large regional decreases in cortical surface area correlate inversely with mean cortical thickness.

The biomechanical forces of brain growth are thought to form the expansion of the cranium so that the skull grows in harmony with the expanding brain^55^. Thus, the positive copy number dosage effect on cortical surface area may directly trigger the effect on head circumference^19–21^ and ICV of 1q21.1 distal carriers due to modifications in pressure. Altered mechanical pressure might also cause the negative copy number dosage effect on the hippocampus and caudate volumes, effects on subcortical volumes also observed in a UK Biobank exploratory study on six individuals with a 1q21.1 distal duplication^56^.

### Human-specific genes may affect the cortical surface area and cross-species effects

The positive copy number dosage effect on brain structure with the same direction as for weight and height^34,35^ likely results from altered gene expression as observed in 1q21.1 distal CNV cell lines^48^. In an independent experiment on fetal tissue, we also observed dynamic expression patterns of the genes in the 1q21.1 interval consistent with potential roles in cortical neurogenesis and development (Supplementary Note 7 and Supplementary Figs. 5 and 6).

GWAS based on the hg19 genome assembly have not identified hits in the 1q21.1 genomic region for ICV^57^, total cortical or regional surface area^53,58^. Assembly of the 1q21.1 region^59^ and thus gene discovery is complicated due to the presence of numerous low copy number repeats^20,43^ and has been faulty until the GRCh38 genome assembly. This may explain the lack of GWAS hits in the region.

Candidates for a dosage-dependent amplifier of the CNV-associated brain phenotypes are the recently identified human-specific *NOTCH2NL* genes that confer delayed neuronal differentiation and increased progenitor self-renewal^22,23^—in line with the radial unit hypothesis^52^. The areas with the highest regional effect sizes overlap with the areas of the highest expression of NOTCH2NLA and C in utero^22^ in concordance with an early developmental effect such as the macrocephaly observed in utero in a 1q21.1 distal duplication carrier^38^. Our observations of a 2% reduced skull diameter in the 1q21.1 deletion mouse (Supplementary Fig. 7 and Supplementary Notes 8 and 9) and recent findings of decreased total brain volume focused on the temporo-parietal and subcortical areas in the deletion mouse^60^ suggest that genes overlapping between human and mice (nine of ten mice genes are syntenic to the human region^42^) and not specific to humans are also involved in the altered skull and brain morphology. However, although diameter and volume are not directly comparable, the 17% decrease in ICV in human 1q21.1 deletion carriers would still point towards a substantial role of human-specific genes or genes with altered functions in comparison to mice. This underlines the need for additional data to disentangle which specific genes are involved in the skull and brain structural phenotypes. Of note, we also observed shorter bones overall in the 1q21.1 deletion mice (Supplementary Fig. 8 and Supplementary Note 9), expanding on previous head-to-tail length data^42^, and lower bone mineral density in female mice (Supplementary Fig. 9 and Supplementary Note 9), which mirror bone characteristics from human deletion carriers^34^ increasing the number of observed cross-species effects between the 1q21.1 mice and human 1q21.1 deletion carriers.

### 1q21.1 distal CNV deletion and duplication carriers show deficits in different cognitive functions

Our findings of widespread lower performance across several tests in different domains for both carrier groups in the volunteer-based UK Biobank sample are in line with cognitive results from a recent study^33^ and support that cognitive function in CNV carriers largely without a neurodevelopmental diagnosis may still be compromised^8,16^. Interestingly, the frontal and cingulate regions^61^, with the greatest cortical effect sizes for distal 1q21.1, correlate particularly with cognitive function and have gone through the greatest expansion during human development and evolution^62^. Our analyses indicated that the decreases in cognitive task performance are partially mediated by the observed differences in ICV and cortical surface area, reflecting the positive correlation between brain volume and intellectual function in line with previous findings^63^. The decrease in performance for several cognitive tasks in duplication carriers despite a larger ICV and cortical surface area suggests that the positive correlations may only be applicable within a certain narrower range. Interestingly, recent genetic analysis of *NOTCH2NL* in archaic and modern humans revealed ongoing adaptive evolution towards a lower dosage of the protein^64^, suggesting negative effects of excessive NOTCH2NL protein.

Our brain structural findings in 1q21.1 distal CNV carriers overlap with brain alterations in associated disorders: for example, ADHD^65^, autism spectrum disorders^66^, schizophrenia^67^, bipolar disorder^68^, major depressive disorder^69^ and subtypes of epilepsy^70^, but the exact overlaps differ between carrier groups. Of note, 1q21.1 distal deletion and duplication carriers display direct, opposite effects on several brain structures, while at risk for the same neurodevelopmental diseases. Other pathogenic CNVs also display overlapping disease risk and similar opposite copy number effects^6,8–15^ including effects on the cortical surface area in 22q11 and 16p11.2 proximal CNV carriers^6,12–14^. These CNVs impact different genes, but may converge on the same downstream pathways altering cortical surface area formation, similar to what has been reported for behavioural and neurocognitive phenotypes^28^.

This also suggests that other risk factors interplay to cause disease. It also supports that subgroups within neurodevelopmental disorders can be defined based on genetic profile and brain structural differences.

We demonstrate large effects of 1q21.1 distal CNVs on brain structure and cognition in humans including a mediation effect. These findings provide insight into molecular mechanisms involved in critical stages of human brain development and mapping of gene dosages to brain structural fingerprints.

## Supplementary information

Supplementary figures and notes

Supplementary tables

## Data Availability

The authors declare that the data supporting the findings of this study are available within the paper and its Supplementary information files. The data were gathered from various resources, and material requests will need to be placed with individual PIs. I.E.S. can provide additional detail upon correspondence. Data from PING are available at NIMH Data Archive: https://ndar.nih.gov/edit_collection.html?id=2607
