# Supplementary material for: 1q21.1 distal copy number variants are associated with cerebral and cognitive alterations in humans
Source: Transl Psychiatry. 2021 Mar 22;11:182. doi: 10.1038/s41398-021-01213-0 (PMC7985307; doi:10.1038/s41398-021-01213-0)
Supplement: Supplementary file 1 — Supplementary figures and notes [file 41398_2021_1213_MOESM1_ESM.docx]

**Supplementary Figures and Notes for: “1q21.1 distal copy number variants are associated with** **cerebral and cognitive alterations in humans”**

**Overview, Supplementary Figures (pages 3-12):**

**Supplementary Figure 1:** Age distribution per cohort contributing data to the current study, with age in years on the y-axis and cohort name on the x-axis.

**Supplementary Figure 2:** Coverage of the 1q21.1 distal region by genotyping platforms in ENIGMA-CNV.

**Supplementary Figure 3:** Bivariate plot of age (years) versus uncorrected ICV (mm3).

**Supplementary Figure 4.** Forest plots on the dosage effect of copy number on subcortical volumes, surface area, thickness and ICV.

**Supplementary Figure 5:** Expression peak of the genes encoded in the 1q21.1 interval during human fetal corticogenesis.

**Supplementary Figure 6:** RNA-seq profile of genes in the 1q21.1 interval during human corticogenesis.

**Supplementary Figure 7**: Skull diameter in 1q21.1 deletion knockout mice in comparison to wildtype (WT) littermates.

**Supplementary Figure 8:** Body weight and bone size of 1q21.1 deletion mice in comparison to wildtype (WT) littermates.

**Supplementary Figure 9:** Bone mass measurements in 1q21.1 mice and wildtype (WT) litter mates.

**Overview, Supplementary Notes (pages 13-20):**

**Supplementary Note 1:** Extended information on datasets.

**Supplementary Note 2:** Details on CNV calling and QC.

**Supplementary Note 3:** Extended information on UK biobank CNV calls. **Supplementary Note 4:** Extended info on image acquisition and processing.

**Supplementary Note 5:** Description of additional sensitivity and robustness analyses.

**Supplementary Note 6:** Details on cognitive task data processing

**Supplementary Note 7:** Details on human fetal transcriptional data

**Supplementary Note 8:** Df(h1q21)+/- mouse characterization

**Supplementary Note 9:** Results on the 1q21.1 distal deletion mouse

**Overview, Supplementary tables, legends (pages 21-26):**

**(NOTE – this is ONLY legends - please refer to separately submitted excel sheet for the entire tables)**

**SUPPLEMENTARY FIGURES:**


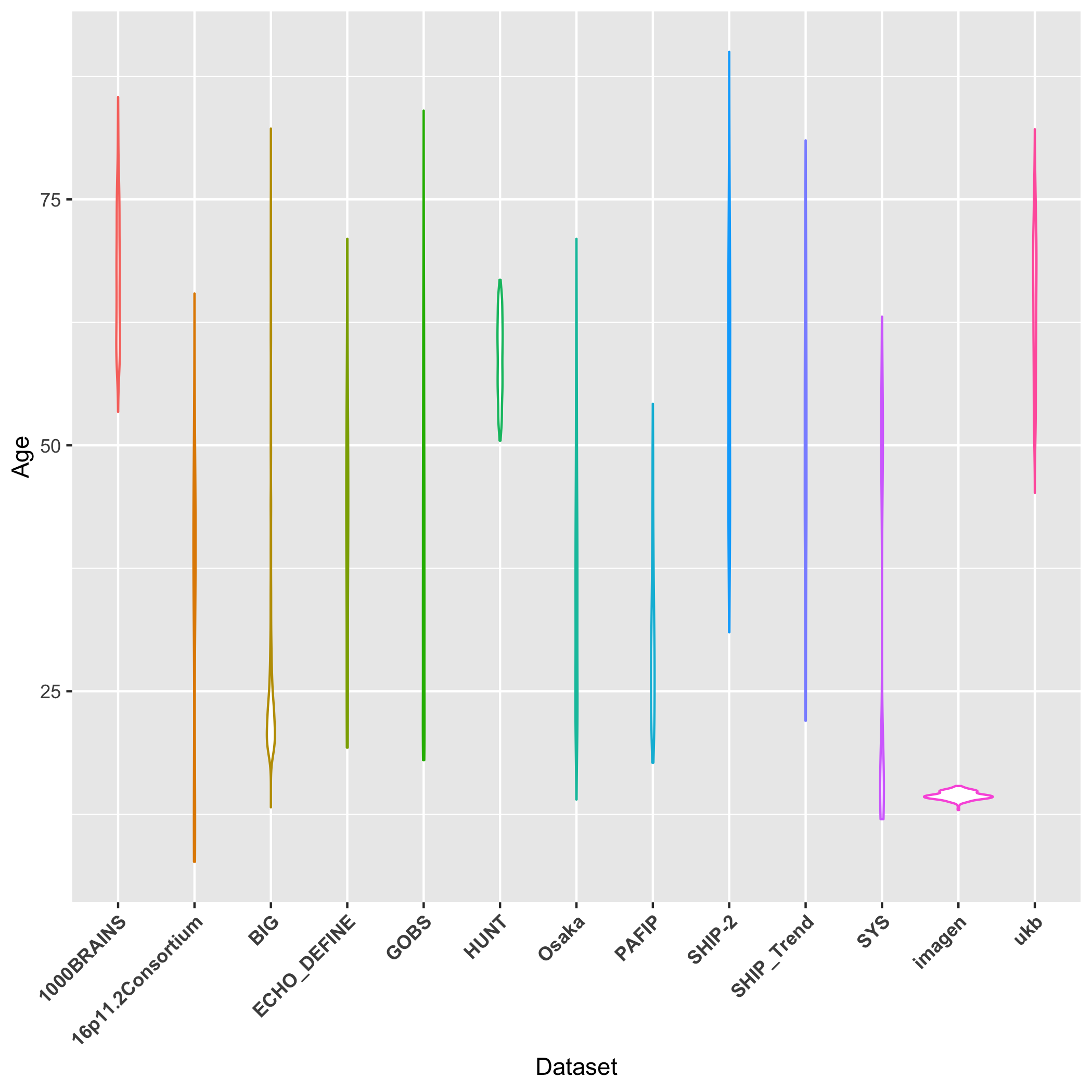


**Supplementary Figure 1: Age distribution per cohort contributing data to the current study, with age in years on the y-axis and cohort name on the x-axis.**


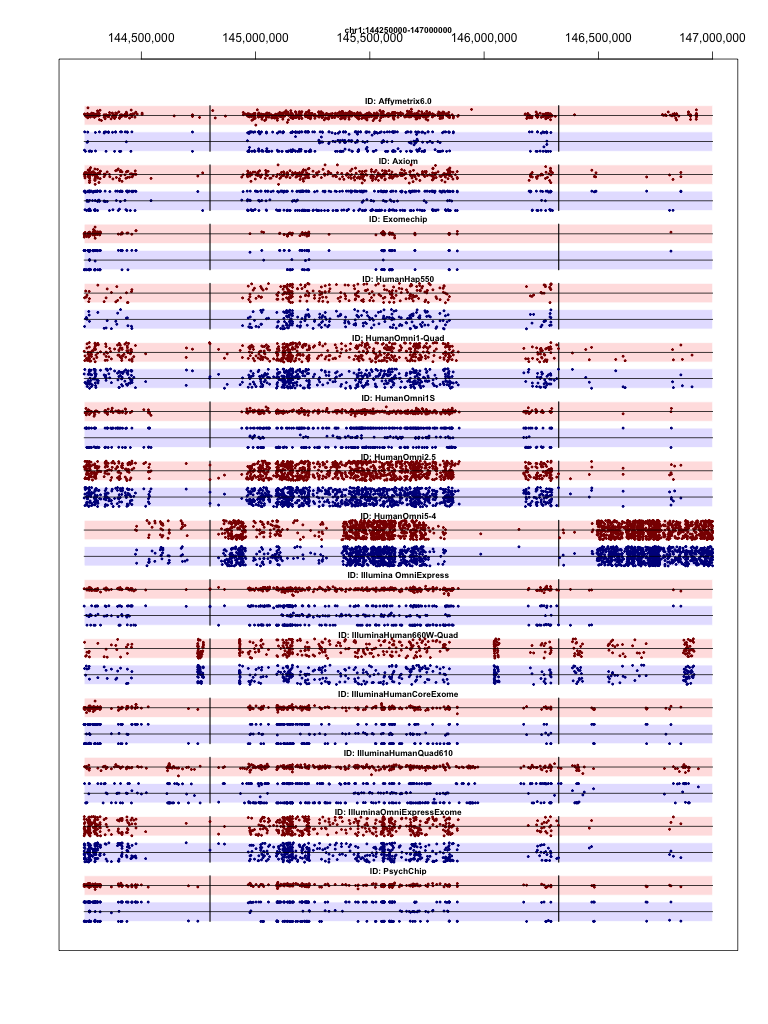
**Supplementary Figure 2: Coverage of the 1q21.1 distal region by genotyping platforms in ENIGMA-CNV.** Log R ratio is shown in red, B-allele frequency in blue. The vertical black lines delimit the boundaries of the 1q21.1 distal region. HumanHap550, HumanOmniQuad1-Quad, HumanOmni2.5, HumanOmni5-4, IlluminaHuman660-Quad, IlluminaOmniExpressExome are mock data. The rest is based on real data.

**Supplementary Figure 3: Bivariate plot of age (years) versus uncorrected ICV (mm3).** Deletion carriers in red, non-carriers in grey and duplication carriers in blue, respectively. Circles = females, triangles = males.

**Supplementary Figure 4. Forest plots on the dosage effect of copy number on subcortical volumes, surface area, thickness and ICV.** The effect size (β of the linear regression) at each site for each measure is shown by the position on the x-axis. Standard error is shown by the horizontal line. A summary polygon shows the results when fitting a random-effects model to the two datasets: the main and the Icelandic deCODE samples. del, nc and dup denote the number of individuals in each analysis. This number changes on the basis of quality control for each structure. * = P < 0.0014, ** = P < 0.00014. Effect size and confidence intervals are to the right.

**Supplementary Figure 5: Expression peak of the genes encoded in the 1q21.1 interval during human fetal corticogenesis.** The highest expression value for each gene in the developmental stages from GW7 to GW21 is indicated. The genes are ordered according to their chromosomal positions. cFPKM = corrected Fragment Per Kilobase and Million reads.

**Supplementary Figure 6: RNA-seq profile of genes in the 1q21.1 interval during human corticogenesis.** The expression value for each gene in corrected Fragment Per Kilobase and Million reads (cFPKM) in the developmental stages from gestation week 7 (GW7) to 21 (GW21) is indicated. The GW21 stage samples include dissection into frontal, temporal, occipital and parietal lobes. The parietal area was further microdissected into the cortical plate (CP) and underlying domains of the cortical wall (non-CP, containing mostly outer-subventricular zone (oSVZ) and ventricular zone germinal zones.

**
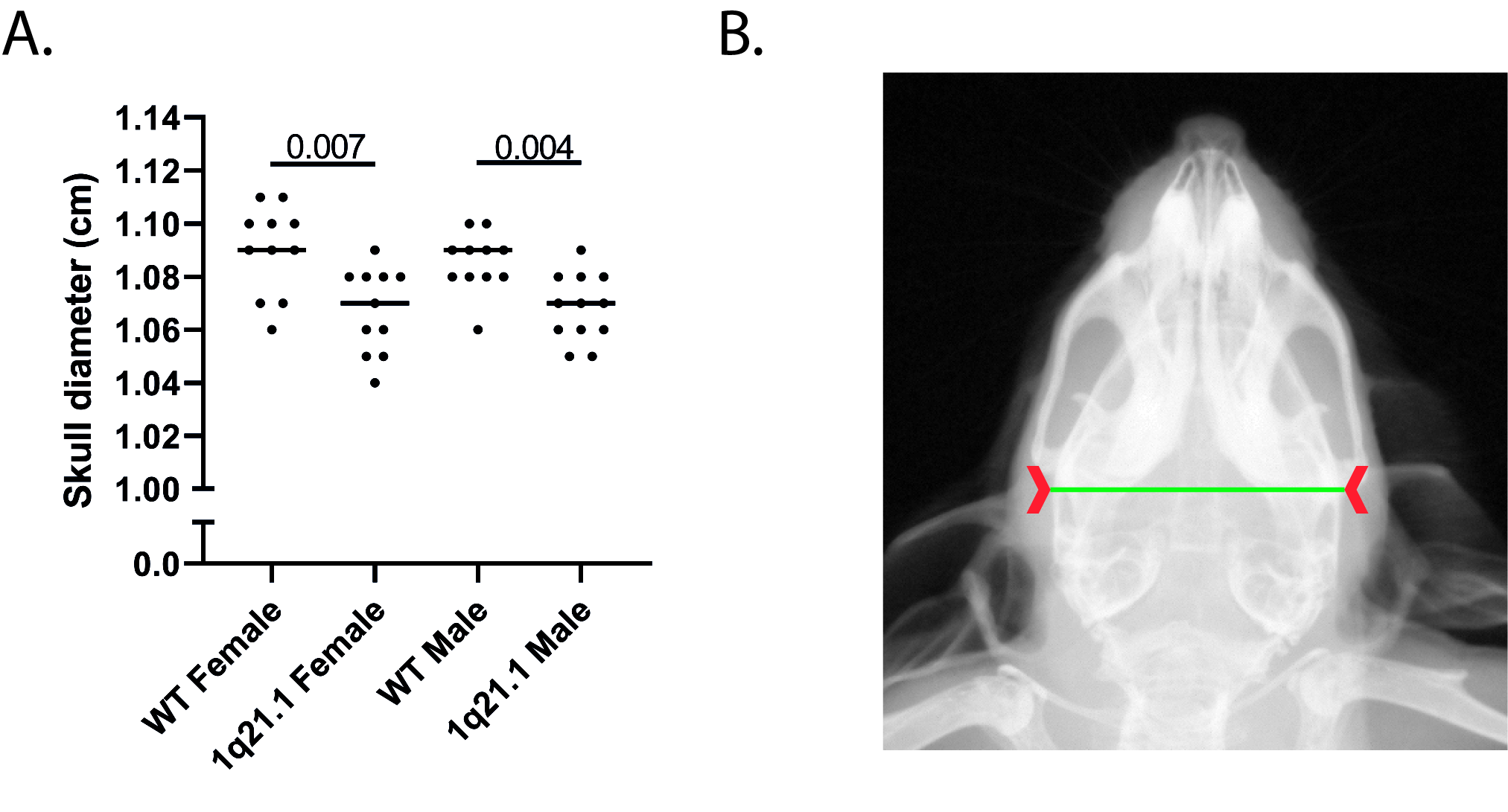
Supplementary Figure 7: Skull diameter in 1q21.1 deletion knockout mice in comparison to wildtype (WT) littermates.** A. Median skull diameter (n=10-12 per group). The horizontal lines demark P-values as group-wise comparisons (non-parametric Mann-Whitney U test) between the genotype groups. B. X-ray showing the mouse skull with the green line indicating how the skull diameter was determined.


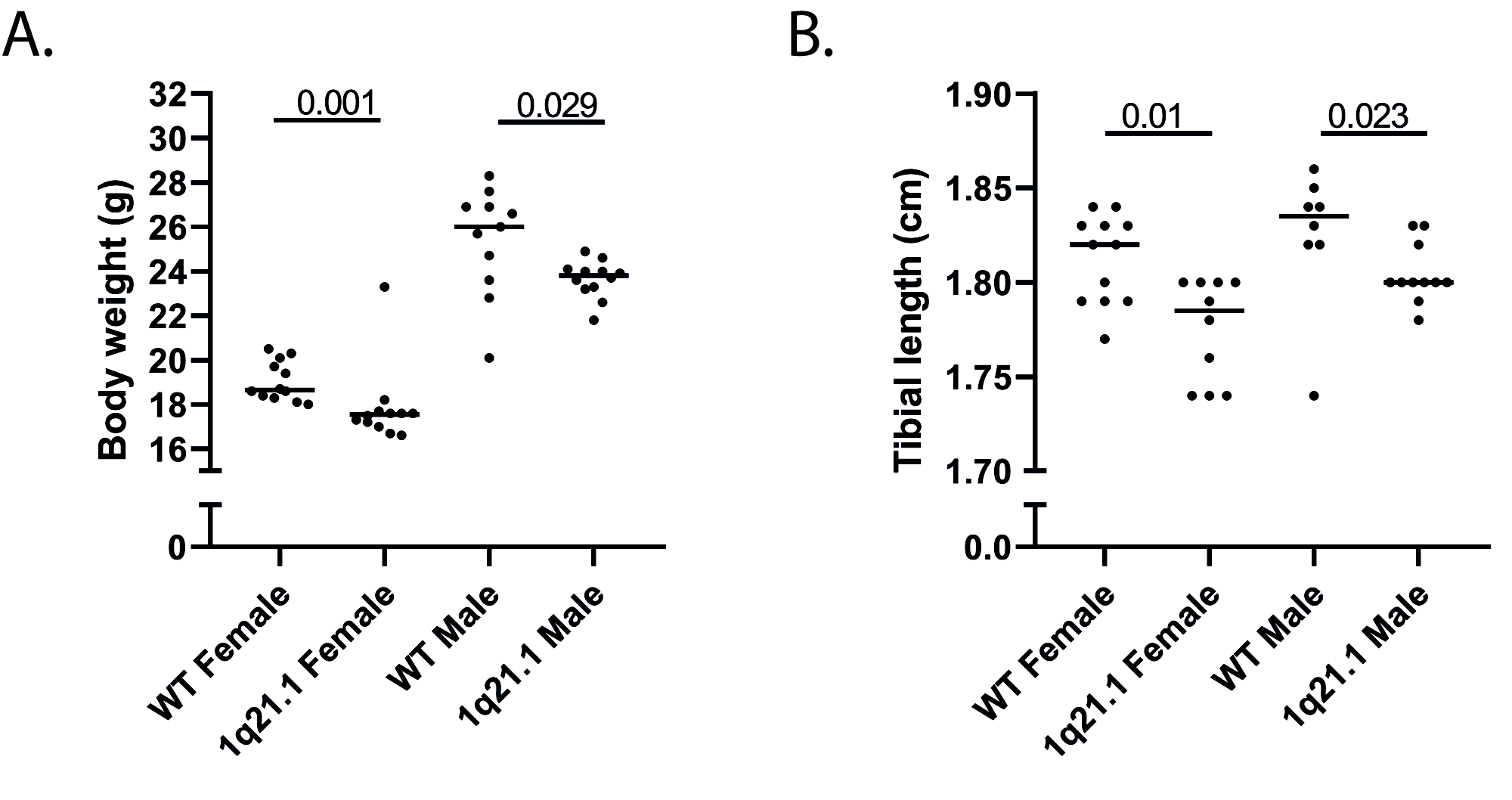
**Supplementary Figure 8: Body weight and bone size of 1q21.1 deletion mice in comparison to wildtype (WT) littermates**. A. Median body weight. B. Tibial (lower leg) length measured on X-rays. P-values show group-wise comparisons (non-parametric Mann-Whitney U test) between the genotype groups (n=10-12 in each group).

**Supplementary Figure 9: Bone mass measurements in 1q21.1 mice and wildtype (WT) litter mates.** Median bone mineral density (BMD) in femur (upper leg) (A) and whole body (D). Median bone mineral content (BMC) in femur (B) and whole body (E). Bone area in femur (C) and whole body (F). P-values show group-wise comparisons (non-parametric Mann-Whitney U test) between the genotype groups.

**SUPPLEMENTARY NOTES**

**Supplementary Note 1: Extended information on datasets and test for differences in demographics.**

Diagnosis-information in ENIGMA-CNV was based on information from the different datasets. In the UK biobank, diagnosis was extracted as Datafield 41202: Diagnoses - main ICD10 and 41204 Diagnoses - secondary ICD10. If either of these contained an F (mental) or G (neurological) disorder, these were coded as affected and the ICD10 disorder was noted.

For the core ENIGMA-CNV dataset, family information was based on pi-hat estimated for pairs of individuals, and only one relative (if more than two) from pairs with pi-hat >0.2 was kept. CNV carriers were selectedly kept over non-carriers. For ECHO_DEFINE and the 16p11.2 European Consortium, relatedness was based on information from the clinican. For the UK biobank, relatives were extracted from Datafield 22011: Genetic relatedness pairing and Datafield 22012: Genetic relatedness factor. One of each pair with a kinship coefficient above 0.053 (that is more related than 1^st^ cousins) was removed.

Tests for differences between groups for demographic data applied a test included in the R package tableone v0.7.3 – chi square test with continuity correction for categorical values and ANOVA for continuous variables.

**Supplementary Note 2: Details on CNV calling and QC.** All PFB-files were based on Human Genome Build NCBI36/hg18 except for UK biobank, ECHO-DEFINE and parts of 16p11.2 European Consortium that used NCBI37/hg19. PFB-and GC-files were selected based on publicly available data from the PennCNV homepage or self-generated: in the case of cohorts primarily consisting of Asian and African individuals, a PFB-file was generated through PennCNV compile_pfb.pl using all genotyping arrays from the cohort. The PFB-file used is noted in Supplementary Table 10.

The following quality control metrics were used: Adjacent CNVs separated by a gap less than 20% of the combined length of the two CNVs were merged until no more gaps of <20% existed, and CNVs based on less than 15 SNPs were excluded. Only samples with standard deviation (SD) of normalized intensity (LRR) <0.35, B allele frequency (BAF) drifting value <0.01 and wave factor value between -0.05 and 0.05 were included.

The 1q21.1 distal region was well-covered by all arrays (Figure S8). CNVs overlapping the region of interest (1q21.1 distal and 1q21.1 distal and proximal) were identified with the R package iPsychCNV SelectSamplesFromROI with parameters OverlapMin = 0.4 and OverlapMax = 5, visualized with iPsychCNV StackPlot and manually inspected. None of the 1q21.1 distal carriers carried additional genomic imbalances (Supplementary Table 1) except for three duplications that extended into the 1q21.1 proximal region (Supplementary Table 2), a known susceptibility factor for thrombocytopenia-absent radius (TAR) syndrome^1^. In the statistical analysis, individuals with a minimum overlap of 0.4 to regions with known pathogenic CNVs (Table S10) were excluded regardless of copy number status as were individuals from scanner sites without 1q21.1 distal CNV carriers.

Carriers in the 16p11.2 European consortium cohort were identified based on report from the cytogeneticist who did the genetic test in the clinic and was thus based on either CGH array or FISH (Fluorescent In Situ Hybridization) - the identification method for each individual carrier is noted in Supplementary Table 2. Non-carriers in the 16p11.2 European consortium cohort were either selected from the general population (excluding individuals with a neurodevelopmental or psychiatric diagnosis) or familiar controls who tested negative for the 1q21.1 distal and proximal CNV or familial controls from a 16p11.2 proximal and distal CNV study - five of the latter had a neuropsychiatric diagnosis. Carriers in the ECHO_DEFINE were identified based on the report from the cytogenetist after genetic test in the clinic with Psych Chip.

**Supplementary Note 3: Extended information on UK biobank CNV calls.**

Anonymised genotyped data was downloaded as l2r & baf-files from UK biobank showcase for chromosomes 1-22, X, Y, M & XY. In addition, snp-files were downloaded. They were stored and processed on a secure Unix server.

For the initial steps, the l2r- and baf-files were split into separate files for each individual containing both l2r and baf-values in 20 batches, each containing 25,000 individuals per batch [the last batch contained 13,377). Subsequently, SNP-names were added to the files. CNVs were called in subbatches of 1000 individuals per batch using PennCNV ^57^ and self-generated PFB- and GCC-model files (NCBI37/hg19) and affygw6.hmm. Subsequent filtering and visualization was done as for the main dataset above except that the LRR_SD cut-off was set at 0.50 given that we observed reliable CNV calls within these ranges. We did not filter based on number of CNVs or genotype call rate. These are quite relaxed filtering criteria but since all 1q21.1 CNVs were visualized and inspected and thus filtered for false positives, we did not apply more stringent parameters. 59 individuals were excluded from the entire UK biobank using these criteria.

**Supplementary Note 4: Extended info on image acquisition and processing.** Each site contributed volumes for the left and right hemispheres of the accumbens, caudate, putamen, pallidum, amygdala, hippocampus and thalamus in addition to right and left 34 regional cortical surface areas and 34 cortical average thicknesses, total surface area and total mean cortical thickness as well as estimated intracranial volume (ICV). The total volume, surface area or mean thickness of each structure was calculated by adding the left and right together. We excluded each individual measure if it deviated more than +/- 4SD from the mean for each individual scanner site.

**Supplementary Note 5: Description of additional sensitivity and robustness analyses.**

We re-analysed the dataset in the following way: (a) MATCHED analysis: Matching each CNV carrier with one non-carrier. The R package Matchit v2.4 was used to match each CNV carrier with one non-carrier based on sex, age, scanner site and ICV. (b) NON-AFFECTED only analysis: Keeping only non-affected individuals (i.e. individuals without a known diagnosis of a brain disorder), (c) NON-AFFECTED ADULTS analysis: Keeping only non-affected adults (age>=18) (d) ADULTS analysis: Only including adults with age>=18 (e) CHILDREN analysis: Only keeping children with age<18, or (f) ENIGMA-CNV ONLY: Keeping only ENIGMA-CNV derived individuals in analysis or (g) UK biobank ONLY: Keeping only UKB-derived participants or (h) POPULATION STRUCTURE analysis: Controlling for population structure by including 4 genetic principal components as covariates calculated based on standardized multidimensional scaling analyses of genome-wide genotype data conducted at each site (i) NO ICV model analysis: Excluding ICV as covariate or j) (f) INCLUDING RELATIVES analysis: Including all relatives (first- or second-degree relatives) that was removed in the primary analysis.

**Supplementary Note 6: Details on cognitive task data processing**

The *Pairs Matching* task (field 399), tested episodic memory, with six pairs of cards being shown for three seconds to participants, before being turned over, after which the participants were asked to identify the matching pairs. We used the total number of errors made. The *Reaction Time* task (field 20023), tested simple processing speed through twelve rounds of a game where participants had to click a button as quickly as possible when shown two matching cards. We used the mean reaction time. *Fluid Intelligence* (field 20016), tested reasoning and problem solving through thirteen verbal and numerical reasoning questions, which had to be answered within two minutes. We used the total number of correct answers. The *Digit Span* task (field 4282) tested numeric working memory by presenting progressively longer numbers to participants and asking them to recall these once the number had disappeared. We used the maximum number of digits correctly recalled. The Symbol Digit Substitution task (field 20195) tested complex processing speed through the matching of numbers to a set of symbols. We used the number of correct substitutions. The Trail Making A and B tasks (fields 20156 and 20157) tested visual attention by asking participants to connect scattered circles according to numbers (trail A) and to alternating numbers and letters (trail B). We used the time taken to complete these tests for our analyses. All data was recoded so that higher scores indicate higher performance.

**Supplementary Note 7: Details on human fetal transcriptional data**

Human fetal tissue collection and preparation was done as described previously^19^ - human fetuses were obtained following medical pregnancy termination. Fetuses aged 7 gestational weeks (GW) (2 males), 9 GW (1 male, 1 undetermined), 12 GW (1 female, 1 undetermined), 15 GW (1 male), and 21 GW (1 male) were used for the RNA sequencing and *in situ* hybridization of cortical tissue. All cases were examined with standard feto-pathological procedures and none displayed clinical or neuropathological evidence of brain malformation. The brain was removed within 6 hours of expulsion and RNA extracted and cDNA prepared^2^. The 350-700bp size cDNA fraction was sequenced from both ends using Hiseq 2500 Rapid mode v3 (Illumina). Transcriptome analysis was performed as previously described^2^. Expression values are calculated as the unit Fragment per kilobase and million reads (FPKM) and those for human-specific duplicated genes (*NBPF10, NOTCH2NLA, HYDIN2, NBPF12, LOC728989, NBPF11, NBPF14, and NOTCH2NLB*) are corrected on the basis of the computer simulation performed in the previous study (cFPKM; corrected FPKM)^2^. The study was approved by three relevant Ethics Committees (Erasme Hospital, Université Libre de Bruxelles, and Belgian National Fund for Scientific Research FRS/FNRS) on research involving human subjects. Written informed consent was given by the parents in each case.

**Supplementary Note 8: Df(h1q21)+/- mouse characterization**

16-week-old male and female heterozygous 1q21.1 deletion knockout mice^3^ and wildtype mice were sacrificed for bone analysis (n=10-12 in each genetic group). Body weight was recorded. Femur and tibia were collected and stored in ethanol at 4 C and in saline at -20 C until further analysis. Animals were genotyped from Taconic (Ejby, Denmark) and genotyping was repeated on tail samples collected after sacrifice. Whole body DXA scans were obtained using a Piximus densitometer (GE Lunar, Madison, WI, USA). Whole-body and femoral bone mineral density (BMD), bone mineral content and bone area were analysed. *X*-ray scans of the skull, upper limbs and lower limbs were obtained using a Faxitron MX-20 small animal x-ray system (Faxitron, Tucson, AZ, USA). Skull diameter, femur length and width, and humerus and tibia length were measured using the ruler function in a dicom viewer program. Measurements were done by staff blinded to genotype groups. 1q21 and wildtype mice were compared using non-parametric Mann-Whitney U test. To take variation in bone turnover between sexes into account, males and female mice (10-12 mice in analytical group) were analyzed separately. Differences were considered significant at P<0.05. All animals were included in the analyses.

**Supplementary Note 9: Results on the the 1q21.1 deletion mouse**

In a comparison between 1q21.1 deletion mice (Df(h1q21) +/- mouse^3^)) and their wild-type littermates (n=10-12 mice per group), we found a significant decrease in skull diameter in the deletion mice (2% decrease, P=0.007 (females) and P=0.004 (males)) (Figure 4). Also, the deletion mice displayed lower weight and shorter tibial (lower leg) length (P=0.01 (females), P = 0.023 (males)) (Supplementary Figure 4). Finally, bone mineral density (BMD), bone mineral content (BMC) and bone area were lower in female 1q21.1 deletion mice compared to wild-type littermates (P<0.0005 (BMD and BMC) and P=0.004 (area)) whereas male deletion mice - unlike the deletion females - displayed an increased femoral bone (upper leg) area (P=0.022; Supplementary Figure 5).

**References:**

1. Albers CA, Paul DS, Schulze H, Freson K, Stephens JC, Smethurst PA *et al.* Compound inheritance of a low-frequency regulatory SNP and a rare null mutation in exon-junction complex subunit RBM8A causes TAR syndrome. *Nat Genet* 2012; **44**(4)**:** 435-S432.

2. Suzuki IK, Gacquer D, Van Heurck R, Kumar D, Wojno M, Bilheu A *et al.* Human-Specific NOTCH2NL Genes Expand Cortical Neurogenesis through Delta/Notch Regulation. *Cell* 2018; **173**(6)**:** 1370-1384.e1316.

3. Nielsen J, Fejgin K, Sotty F, Nielsen V, Mork A, Christoffersen CT *et al.* A mouse model of the schizophrenia-associated 1q21.1 microdeletion syndrome exhibits altered mesolimbic dopamine transmission. *Translational psychiatry* 2017; **7**(11)**:** 1261.

**SUPPLEMENTARY TABLES LEGENDS:**

**(NOTE – this is ONLY legends - please refer to separately submitted excel sheet for the entire tables)**

**Supplementary Table 1: Specification of all cohorts in ENIGMA CNV.** Study design, participant demographics, and references to articles containing descriptions of individual inclusion and exclusion parameters for all datasets in ENIGMA-CNV collected up until Sep 30 2019. Data sets contributing data to the 1q21.1 distal analysis are marked with a star.

**Supplementary Table 2: CNVs of Interest.** Individuals with a minimum overlap of 0.4 to these CNVs were excluded from the analysis. Coordinates are Human Genome Build NCBI36/hg18 and GRCh37/hg19.

**Supplementary Table 3: Chips and corresponding PFB-files used for PennCNV CNV calling.**

**Supplementary Table 4:** **Technical details concerning scanners and acquisition parameters utilized at the participating ENIGMA-CNV scanner sites**

**Supplementary Table 5: Sensitivity analyses – dosage effect of 1q21.1 distal copy number on subcortical volumes in the main sample.**  The effect size (β of the linear regression) is presented with 95 % confidence intervals. A linear regression based on the copy number state of the individuals (deletion=1, normal=2, duplication=3) was performed on normalized brain measures corrected for *plusICV*: age, age squared, sex, scanner site and ICV (except for ICV) or *noICV*: age, age squared, sex, scanner site. Analysis was performed on : ALL – all individuals, ADULTS – adults (age ≥18), NON-AFFECTED - individuals without a known diagnosis of a brain disorder, NON-AFFECTED ADULTS – adult individuals without a known diagnosis of a brain disorders, MATCHED CONTROLS - matching each carrier with one non-carrier based on age, sex and scanner site or matching each carrier with one non-carrier based on age, sex, scanner site and ICV, POPULATION STRUCTURE – checking effect of population structure on individuals. Only individuals with accessible ancestry information were included in the analysis. ENIGMA-CNV ONLY – ENIGMA-CNV dataset exclusively. UKB ONLY – UK biobank dataset only, INCLUDING RELATIVES– including relatives with more than third degree relationships. Results were considered statistically significant if they were below a Bonferroni-corrected P-value of 0.0014. * = P < 0.0014, ** = P < 0.00014, ***=P<0.000014.

**Supplementary Table 6: Sensitivity analyses - T-tests on subcortical volumes between different 1q21.1 distal copy number groups in the main sample.** The effect size (Cohen’s D) including 95 % confidence interval is presented. T-tests were performed on normalized values of brain measures *plusICV*: age, age squared, sex, scanner site and ICV (except for ICV) or *noICV*: age, age squared, sex, scanner site. Analysis was performed on: ALL – all individuals, ADULTS – adults (age ≥18), CHILDREN – children (age<18 years), CHILDREN – children (age<18 years), NON-AFFECTED - individuals without a known diagnosis of a brain disorder, NON-AFFECTED ADULTS – adult individuals without a known diagnosis of a brain disorders, MATCHED CONTROLS - matching each carrier with one non-carrier based on age, gender and scannersite or matching each carrier with one non-carrier based on age, sex, scannersite and ICV, POPULATION STRUCTURE – checking effect of population structure on individuals. Only individuals with accessible ancestry information were included in the analysis, ENIGMA-CNV ONLY – ENIGMA-CNV dataset exclusively. UKB ONLY – UK biobank dataset only, INCLUDING RELATIVES– including relatives with more than third degree relationships. Results were considered statistically significant if they were below a Bonferroni-corrected P-value of 0.0014. * = P < 0.0014, ** = P < 0.00014, ***=P<0.000014.

**Supplementary Table 7: Sensitivity analyses - 1q21.1 distal dosage effect on regional cortical surface area and mean cortical thickness.** The effect size (β of the linear regression) is presented with 95 % confidence interval. A linear regression based on the copy number state of the individuals (deletion=1, normal=2, duplication=3) was performed on normalized brain measures corrected for *plusICV*: age, age squared, sex, scanner site and ICV (except for ICV) or *noICV*: age, age squared, sex, scanner site. Analysis was performed on all individuals with measures available. Analysis was performed on: ALL – all individuals, ADULTS – adults (age ≥18), NON-AFFECTED - individuals without a known diagnosis of a brain disorder, NON-AFFECTED ADULTS – adult individuals without a known diagnosis of a brain disorders, MATCHED CONTROLS - matching each carrier with one non-carrier based on age, gender and scanner site or matching each carrier with one non-carrier based on age, sex, scanner site and ICV, POPULATION STRUCTURE – checking effect of population structure on individuals. Only individuals with accessible ancestry information were included in the analysis NO RELATIVES– excluding relatives with more than third degree relationships. ENIGMA-CNV ONLY – ENIGMA-CNV dataset exclusively. UKB ONLY – UK biobank dataset only, INCLUDING RELATIVES– including relatives with more than third degree relationships. Results were considered statistically significant if they were below a Bonferroni-corrected P-value of 0.0014. * = P < 0.0014, ** = P < 0.00014, ***=P<0.000014.

**Supplementary Table 8: Sensitivity analyses: T-tests on regional cortical surface area and mean cortical thickness.** The effect size (Cohen’s D) including 95 % confidence interval is presented. T-tests were performed on normalized values of brain measures *plusICV*: age, age squared, sex, scanner site and ICV (except for ICV) or *noICV*: age, age squared, sex, scanner site. Analysis was performed on all individuals with measures available. Analysis was performed on: ALL – all individuals, ADULTS – adults (age ≥18), CHILDREN – children (age<18 years), CHILDREN – children (age<18 years), NON-AFFECTED - individuals without a known diagnosis of a brain disorder, NON-AFFECTED ADULTS – adult individuals without a known diagnosis of a brain disorders, MATCHED CONTROLS - matching each carrier with one non-carrier based on age, gender and scannersite or matching each carrier with one non-carrier based on age, sex, scannersite and ICV, POPULATION STRUCTURE – checking effect of population structure on individuals. Only individuals with accessible ancestry information were included in the analysis. ENIGMA-CNV ONLY – ENIGMA-CNV dataset exclusively. UKB ONLY – UK biobank dataset only, INCLUDING RELATIVES– including relatives with more than third degree relationships. Results were considered statistically significant if they were below a Bonferroni-corrected P-value of 0.0014. * = P < 0.0014, ** = P < 0.00014, ***=P<0.000014.

**Supplementary Table 9: Demographic details of ENIGMA-CNV and UK biobank separately.**

**Supplementary Table 10: Extended information on 1q21.1 distal carriers.** Established diagnosis (1 = yes, 0 = no), DiseaseType = known diagnosis or type of study. Chip = genotyping chip used for CNV calling. No of rels = number of known relatives in dataset. Relative = relative in dataset, relative removed = whether individual was removed from the analysis without relatives.

**Supplementary Table 11: Meta-analysis of dosage effect of 1q21.1 distal copy number on subcortical volumes.** The effect size (β of the linear regression) is presented. A linear regression based on the copy number state of the individuals (deletion (del) =1, non-carrier (nc) =2, duplication (dup)=3) was performed on normalized brain measures correcting for age^2^, age, sex and scannersite (and ICV) ) in the ENIGMA-CNV and UK biobank (main sample) and the independent Icelandic cohorts. A final effect size estimate of the combined sample was obtained using a fixed effects meta-analysis framework (metafor). Results were considered statistically significant if they were below a Bonferroni-corrected P-value of 0.0014. * = P < 0.0014, ** = P < 0.00014, ***=P < 0.000014, CI = confidence. Q = statistics for the test for heterogeneity, p(Q) = p-value for the test for heterogeneity, I2 =heterogeneity levels.

**Supplementary Table 12: Meta-analysis of t-tests on subcortical volumes between different 1q21.1 distal copy number groups.** The effect size (Cohen’s D) including 95 % confidence interval is presented. T-tests were performed on normalized values of brain measures correcting for age^2^, age, sex and scanner site (and ICV) in the ENIGMA-CNV and UK biobank (main sample) and the independent Icelandic cohorts. A final effect size estimate of the combined sample was obtained using a fixed effects meta-analysis framework (metafor). Results were considered statistically significant if they were below a Bonferroni-corrected P-value of 0.0014. * = P < 0.0014, ** = P < 0.00014, ***=P < 0.000014, CI = confidence. Q = statistics for the test for heterogeneity, p(Q) = p-value for the test for heterogeneity, I2 =heterogeneity levels.

**Supplementary Table 13: Available sample sizes per task, per carrier group, for the analyses linking the neuroimaging measures to the cognitive measures.** In the analyses, we included all 1q21.1 CNV carriers and non-carriers in the UK Biobank with data on the seven cognitive tasks and brain structures.
